# The experience of the National Institute for Infectious Diseases "Prof. Dr. Matei Balş" in bacterial resettlement therapy trough fecal microbiota transplant in recurrent infections with *C. difficile*

**DOI:** 10.1186/1471-2334-14-S7-O32

**Published:** 2014-10-15

**Authors:** Cătălin Apostolescu, Loredana Benea, Valeriu Gheorghiță, Adina Ilie, Cristina Tenea

**Affiliations:** 1 National Institute for Infectious Diseases "Prof. Dr. Matei Balş", Bucharest, Romania

## Background

The increasing frequency of *C. difficile* infections and the increased frequency of relapses imposed implementation of new treatment alternatives.

## Methods

Starting with July 2013, in the National Institute for Infectious Diseases "Prof. Dr. Matei Balş" we performed 32 therapies in patients with relapsing infections trough bacterial re-colonization.

## Results

We used as donors blood relatives (children, grandchildren) in 68.8% and in 31.2% cases we used stools from others. The success rate at 90 days was 96.8%. This success rate was achieved with a single procedure in 74.1% of cases, with two procedures in 22.5% and in 3.2% of cases with three procedures. The success rate was significantly higher in the first group.

## Conclusion

Although the success of this maneuver is significantly higher than the standard antibiotic treatment, there is need to deepen the experience before generalizing it.

